# Promising Epigenetic Biomarkers Associated With Cancer-Associated-Fibroblasts for Progression of Kidney Renal Clear Cell Carcinoma

**DOI:** 10.3389/fgene.2021.736156

**Published:** 2021-09-23

**Authors:** Yongke You, Yeping Ren, Jikui Liu, Jianhua Qu

**Affiliations:** ^1^ Department of Nephrology, Shenzhen University General Hospital, Shenzhen, China; ^2^ Department of Hepatobiliary Surgery, Peking University Shenzhen Hospital, Shenzhen, China

**Keywords:** kidney renal clear cell carcinoma, cancer-associated fibroblasts, epigenetic regulation, DNA methylation, epigenetic biomarkers

## Abstract

Kidney renal clear cell carcinoma (KIRC) is the most common malignant kidney tumor as its characterization of highly metastatic potential. Patients with KIRC are associated with poor clinical outcomes with limited treatment options. Up to date, the underlying molecular mechanisms of KIRC pathogenesis and progression are still poorly understood. Instead, particular features of Cancer-Associated Fibroblasts (CAFs) are highly associated with adverse outcomes of patients with KIRC, while the precise regulatory mechanisms at the epigenetic level of KIRC in governing CAFs remain poorly defined. Therefore, explore the correlations between epigenetic regulation and CAFs infiltration may help us better understand the molecular mechanisms behind KIRC progression, which may improve clinical outcomes and patients quality of life. In the present study, we identified a set of clinically relevant CAFs-related methylation-driven genes, NAT8, TINAG, and SLC17A1 in KIRC. Our comprehensive *in silico* analysis revealed that the expression levels of NAT8, TINAG, and SLC17A1 are highly associated with outcomes of patients with KIRC. Meanwhile, their methylation levels are highly correlates with the severity of KIRC. We suggest that the biomarkers might contribute to CAFs infiltration in KIRC. Taken together, our study provides a set of promising biomarkers which could predict the progression and prognosis of KIRC. Our findings could have potential prognosis and therapeutic significance in the progression of KIRC.

## Introduction

The global incidence of kidney cancer is increasing. Approximately 400,000 new cases of kidney cancer are diagnosed worldwide in 2018 (Bray et al., 2018; Hoefflin et al., 2020). KIRC is the most prevalent type of kidney cancer with an increasing prevalence (Frew and Moch, 2015). Among kidney cancers, KIRC is the leading cause of cancer-related death, mainly due to its highly metastatic potential and high relapse rate (Kaelin, 2009; Jemal et al., 2011; Jonasch et al., 2012). Meanwhile, KIRC is relatively resistant to traditional chemotherapy and radiotherapy (Jonasch et al., 2012). Therapeutic options for patients with metastatic KIRC are limited, and the prognosis remains dismal. Up to now, there is a lack of biomarkers for diagnosis/prognosis prediction and drug targets for therapeutic intervention of KIRC. The overall prognosis of patients with KIRC is still limited, indicating the need for the improvement of therapeutic strategies directed at potential molecular targets (Li et al., 2017). Thus, it is essential and meaningful to identify reliable new biomarkers for better understand the prognosis and progression of KIRC, and further develop novel therapeutic strategies against KIRC.

Recent studies have emphasized the role of the tumor microenvironment or stromal infiltrates in tumor progression and response to various therapies of tumors. Stromal cell infiltration plays a crucial role in tumorigenesis, progression, metastasis, and clinical outcomes. Cancer-associated fibroblasts (CAFs) are of outstanding importance in tumor stromal infiltration. CAFs are the predominant and critical component in the tumor stromal, and their primary function is to provide a microenvironment for promoting tumor cell characteristics associated with increased aggressiveness. Cancer is associated with CAFs at all stages of cancer progression, including initiation, growth, and metastasis of tumor, and they are considered as a niche response to tissue damage caused by cancer cells. CAFs produce various tumor-associated components and play a role in regulating tumor extracellular matrix, tumor cell metabolism, and immune infiltration of the tumor microenvironment (Kalluri, 2016; Curtis et al., 2019). CAFs were proposed to have a protumor effect in kidney cancer. The study by Xu et al. showed that CAFs were involved in tumor progression by influencing cell proliferation, migration, and drug resistance in kidney tumors (Xu et al., 2015; Errarte et al., 2020). In particular, the symbiotic correlation between tumor cells and CAFs was proposed in KIRC, and CAFs seem to be involved in the initial phases of KIRC progression (Bakhtyar et al., 2013; López, 2013; Errarte et al., 2020). However, resulting from a lack of proper experimental models to study CAFs in KIRC, the role of CAFs in KIRC remains to be further explored.

DNA methylation is one of the widely studied epigenetic modifications (Egger et al., 2004; Feinberg, 2007; Bock and Lengauer, 2008) and plays crucial roles in tumorigenesis and progression across tumors. Furthermore, variation of DNA methylation status has been demonstrated to be associated with clinical features of patients with tumor (Morris and Maher, 2010; Morris and Latif, 2017). Previous studies have proposed that DNA methylation status contributes to progression and clinical outcomes of patients with kidney cancers suggesting that DNA methylation has the potential to be prognostic biomarkers and therapeutic targets for KIRC (Ellinger et al., 2011; Ricketts et al., 2014; Eggers et al., 2012; Patrício et al., 2013; Xiao et al., 2013; He et al., 2013). The aberrant DNA methylations have been shown as independent prognostic markers for kidney cancers (Yamada et al., 2006; Morris et al., 2010; Atschekzei et al., 2012; Van Vlodrop et al., 2017). DNA methylation status was proposed to have the potential to improve outcomes of patients with KIRC as well as diagnosis, prognosis, and clinical treatment of KIRC (Evelönn et al., 2019; Angulo et al., 2021). DNA methylation studies relevant to KIRC to date are still limited. No study has reported DNA methylation in CAFs-related genes as prognostic markers for patients with KIRC.

In the present study, we sought to investigate the potential role of CAFs-related DNA methylation genes in clinical outcomes of patients with KIRC. The CAFs-related DNA methylation genes were screened using database-based bioinformatic analysis, and their associations with clinical features were evaluated.

## Methodology

### Dataset Download and Processing

The gene expression, DNA methylation and relevant clinical datasets for human KIRC samples were generated by The Cancer Genome Atlas (TCGA). The RNA expression dataset was obtained from Xena UCSC, containing 530 cases (Goldman et al., 2018). The gene expression dataset of normal kidney tissues was downloaded from GTEx database (The Genotype-Tissue Expression project). The DNA methylation dataset was obtained from TCGA database and arranged using R language.

### Gene Ontology Analysis

GO analysis was conducted according to differentially expressed genes using the R package clusterprofiler in R language. The GO terms included three categories: Biological process (BP), cellular component (CC) and molecular function (MF).

### Stromal and Immune Infiltration Assessment

The stromal and immune infiltrations were evaluated by MCPCounter. Using MCPCounter R package in R, the stromal and immune cell infiltration levels of KIRC samples were calculated.

Eight immune cells and two stromal cells were quantified in human KIRC samples.

### Correlation Analysis

To examine the correlation between DNA methylation status of relevant genes and their RNA expression, we performed correlation analysis using datasets from TCGA database. Spearman’s correlation coefficient were performed to assess the strength of the relationship between two variables. The analysis was carried out using R language.

### Survival Analysis

To examine the clinical significance of DNA methylation status of relevant genes and their RNA expression in KIRC, survival analysis was carried out. For transcriptional regulatory mechanism at epigenetic level, hypermethylation should be accompanied by a reduction of its gene expression. Conversely, hypomethylation should overlap with upregulation of its gene expression. We combined gene expression levels and their methylation status, and then the joint Kaplan-Meier survival analysis of methylation and expression was conducted. Kaplan-Meier analysis was performed and compared using the logrank test. A value of *p* < 0.05 was used to indicate statistical significance. Bioinformatic analyses and statistical analyses were conducted using R language. Survival and survminer packages were used for Kaplan–Meier curves in R language. Survival package was used for computing survival analyses. Survminer package was used for summarizing and visualizing the results of survival analyses.

### Methylation-Driven Gene Screening

The correlations between DNA methylation status of relevant genes and their RNA expression levels were assessed using Spearman’s correlation coefficient. |r | > 0.3 and *p* < 0.05 served as the screening threshold.

### Statistical Analysis

Group comparisons in bioinformatic analysis were carried out by Wilcox test and Kruskal-Wallis test. We performed survival analysis using Kaplan-Meier method with the logrank test. Spearman’s correlation test was performed to examine the correlation coefficients in the study. A value of *p* < 0.05 was used to indicate statistical significance.

## Results

### Evaluation of Clinical Relevance of CAFs in KIRC

**FIGURE 1:**
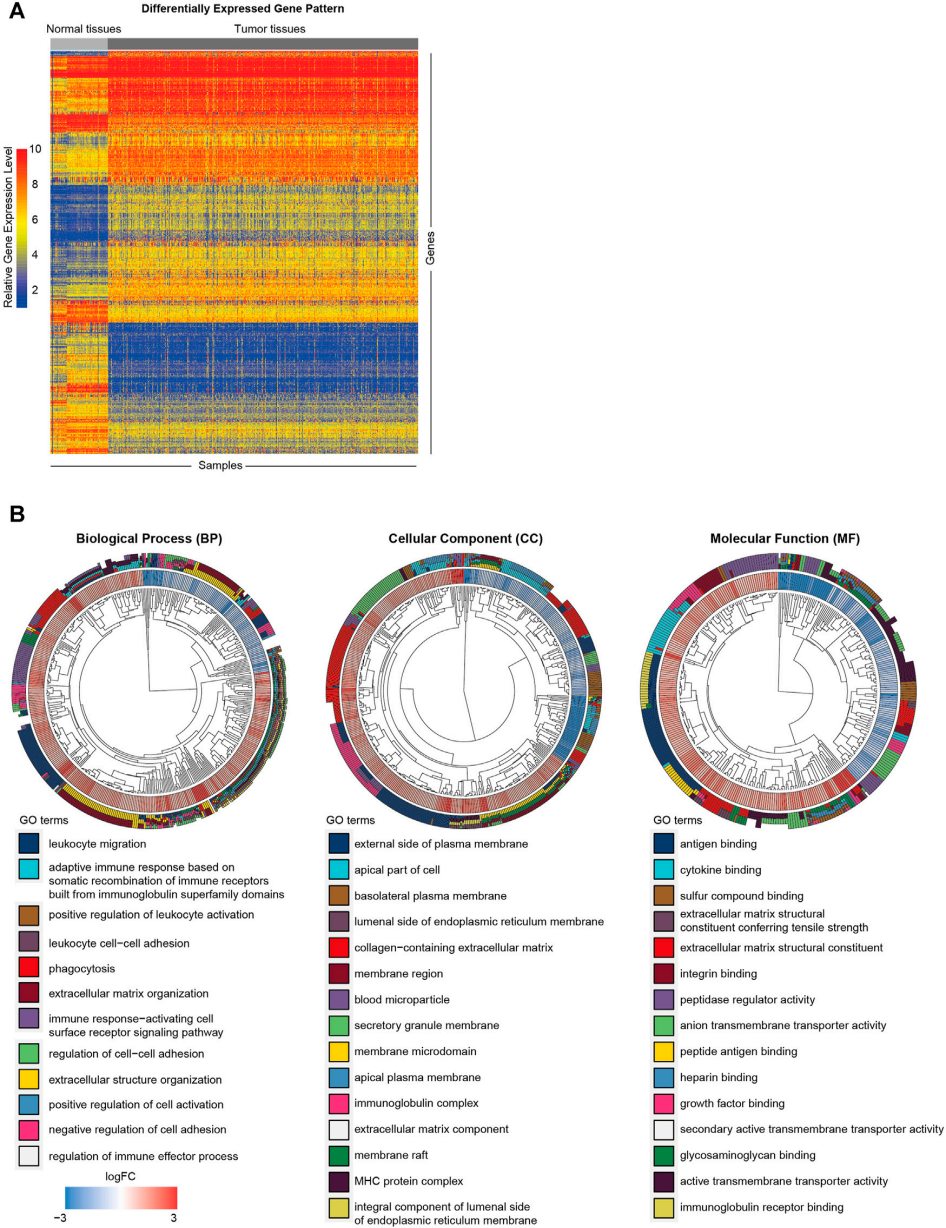
Functional annotation of differentially expressed genes between KIRC tumor and normal tissues. **(A)** Heat map of differentially expressed gene analysis. The ordinate represents the differentially expressed genes while the normal and tumor samples is represented in the abscissa. The blue color indicates lower expression, and the red color indicates higher expression. **(B)** The GO analysis of differentially expressed genes between normal and tumor tissues.

**FIGURE 2:**
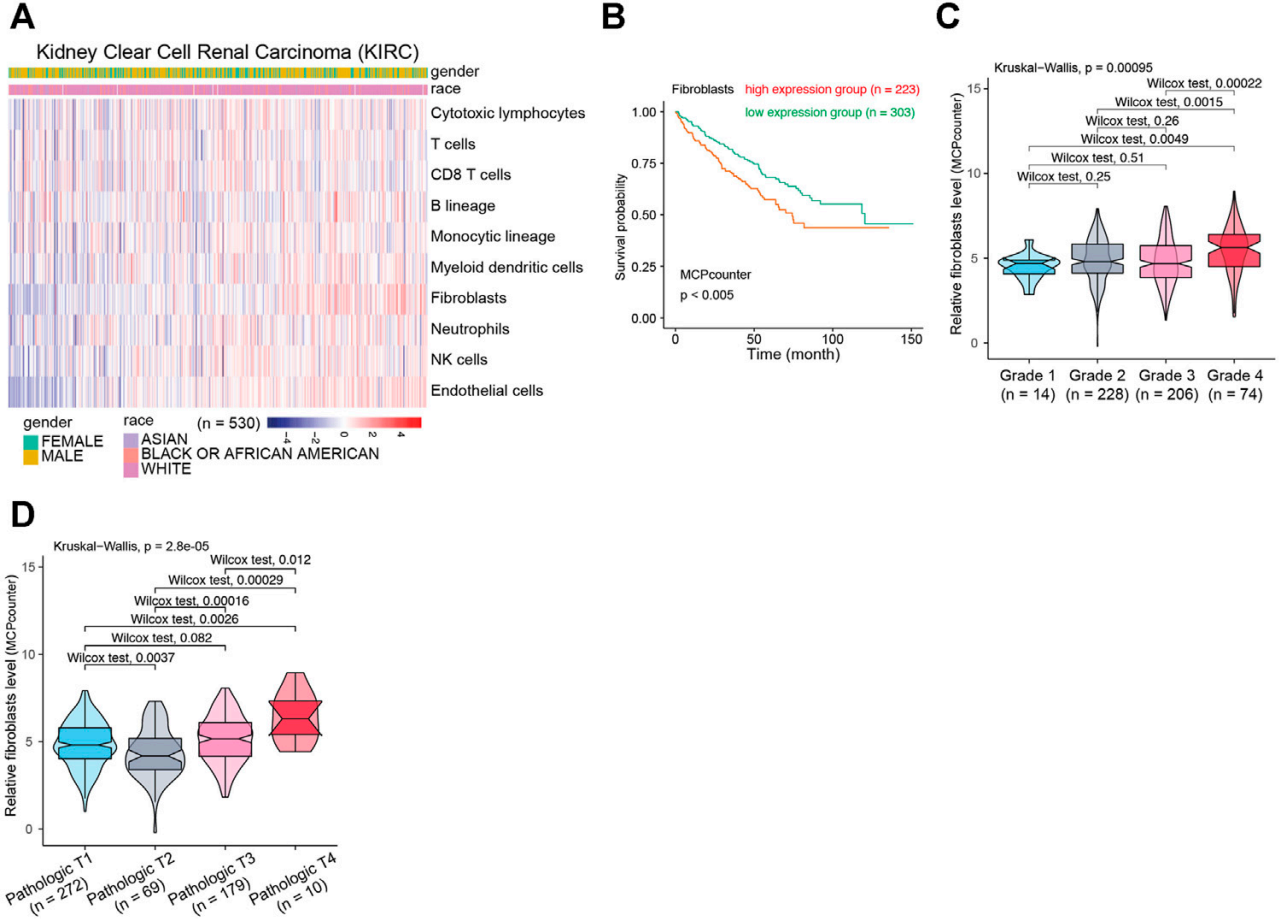
The relationship between CAFs infiltration level and clinical features of patients with KIRC. **(A)** The evaluation of immune and stromal infiltration levels of KIRC based on MCPCounter algorithm. **(B)** Kaplan-Meier survival analysis according to infiltration level of CAFs in KIRC. **(C)** Association between CAFs infiltration level and KIRC histologic grade. **(D)** Association between CAFs infiltration level and KIRC pathological T grade.

To clarify the biological functions of differentially expressed genes (Figure 1A) between normal and tumor tissues of KIRC, differential gene analysis was carried out using datasets from GTEx and TCGA databases. The corresponding relationship table between tumor samples and normal samples was downloaded from the differential analysis section of GEPIA database (http://gepia.cancer-pku.cn/help.html). The TCGA tumor samples, TCGA paired adjacent normal samples and GTEX normal samples were arranged and used for the analysis. Then gene ontology (GO) analysis was performed. The R package clusterprofiler was used for the analysis (Yu et al., 2012). As shown in Figure 1B, we observed that the tumor microenvironment-related terms (such as cell adhesion and extracellular matrix related terms) were significantly enriched in categories Biological process (BP), Cellular Component (CC) and Molecular Function (MF), respectively. These results suggested that tumor microenvironment changes might contribute to KIRC tumorigenesis. The importance of tumor microenvironment sets the basis for our following CAFs relevant study in KIRC. To specifically study the role of CAFs in KIRC, we firstly evaluated the infiltration level of CAFs in KIRC using MCPCounter (Becht et al., 2016). As shown in Figure 2A, the quantification of the abundance of eight immune cells and two stromal cells was performed using KIRC transcriptomic dataset downloaded from the TCGA database. In order to explore the importance of CAFs in KIRC, we performed a series of analyses to examine the relationship between CAFs and clinical features of KIRC. To investigate the correlation between CAFs and survival of patients with KIRC. We then examined the association between CAFs and the survival conditions of patients using Kaplan-Meier survival analysis. The survival analysis showed that the infiltration of CAFs was significantly negatively correlated with the overall survival of KIRC patients (Figure 2B, logrank test). We next investigated the relationship between CAFs and histologic grade. As shown in Figure 2C, a trend of positive correlation was observed between CAFs infiltration level and the histologic grade. Higher infiltration level of CAFs was accompanied by a relative higher tumor histologic grade than lower CAFs infiltration level. Furthermore, we examined the correlation between CAFs infiltration level and tumor stage. The result showed that CAFs infiltration level had a significant positive correlation with tumor T stage, referring to the size and extent of the primary tumor (Figure 2D). Meanwhile, the CAFs infiltration in KIRC was also evaluated using XCell (Aran et al., 2017). Similar outcomes were obtained. We observed a negative correlation between CAFs and survival of patients in Kaplan-Meier analysis (Supplementary Figure S1A), as well as positive correlations between CAFs and tumor histologic grade/tumor T stage (Supplementary Figures S1B,C). Taken together, these findings suggest that increased CAFs infiltration was associated with aggressive clinical features of KIRC. All in all, results demonstrate that CAFs were involved in the progression and development of KIRC. Therefore, CAFs have potential clinical implication for diagnosis, prognosis, and treatment of KIRC.

### Identification of CAFs-Related Methylation-Driven Genes in KIRC

**FIGURE 3:**
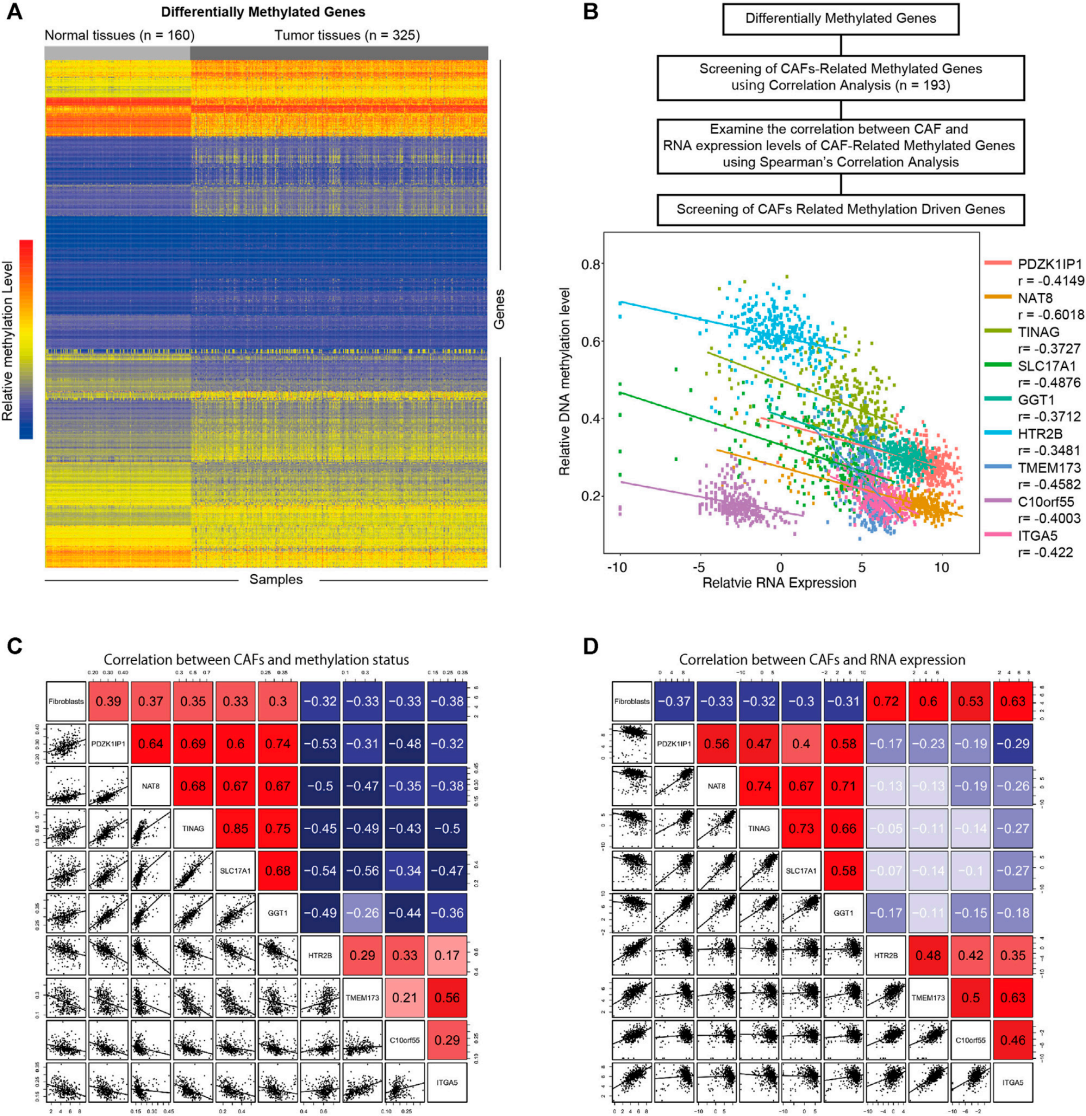
Screening of CAFs related methylation-driven genes. **(A)** Heat map of differentially methylated gene analysis. The ordinate represents the differentially methylated genes while the normal and tumor samples is represented in the abscissa. The blue color indicates lower methylation levels, and the red color indicates higher methylation levels. **(B)** Flowchart of CAFs related methylation-driven genes screening (top). Spearman’s correlation between RNA expression level of CAFs related methylation-driven genes and their methylation status (bottom). **(C)** Spearman’s correlation between CAFs infiltration level and methylation status of CAFs related methylation-driven genes. **(D)** Spearman’s correlation between CAFs infiltration level and RNA expression of CAFs related methylation-driven genes.

To investigate the influence of DNA methylation on CAFs, we firstly identified differentially methylated genes in KIRC (Figure 3A) (|logFC | > 0.2, *p* < 0.05). Through the correlation analysis between methylation status and CAFs infiltration level, we screened the CAFs-related methylated genes. We then examine the correlations between the methylation level of CAFs-related methylated genes and their mRNA expression levels. According to a cutoff value of r > 0.3, *p* < 0.05. We identified nine CAFs-related methylation-driven genes (Figure 3B). Furthermore, the correlation between CAFs infiltration level and the methylation levels, as well as mRNA expression levels of CAFs-related methylation-driven genes, were shown in Figures 3C,D. We observed positive correlations between CAFs infiltration level and methylation status of PDZK1IP1, NAT8, TINAG, SLC17A1, and GGT1. Methylation status of HTR2B, TMEM173, C10orf55, and ITGA5 were negatively correlated with CAFs infiltration level. The correlations between CAFs infiltration level and RNA expression levels of CAFs-related methylation-driven genes were examined. In contrast, RNA expression levels of PDZK1IP1, NAT8, TINAG, SLC17A1, and GGT1 were demonstrated to exhibit negative correlations with CAFs infiltration level. RNA expression levels of HTR2B, TMEM173, C10orf55 and ITGA5 were positively correlated with CAFs infiltration level. Overall, we identified a set of CAFs-related methylation-driven genes that might contribute to the tumor microenvironment via regulating CAFs infiltration.

### Survival Significance of CAFs-Related Methylation-Driven Genes

**FIGURE 4:**
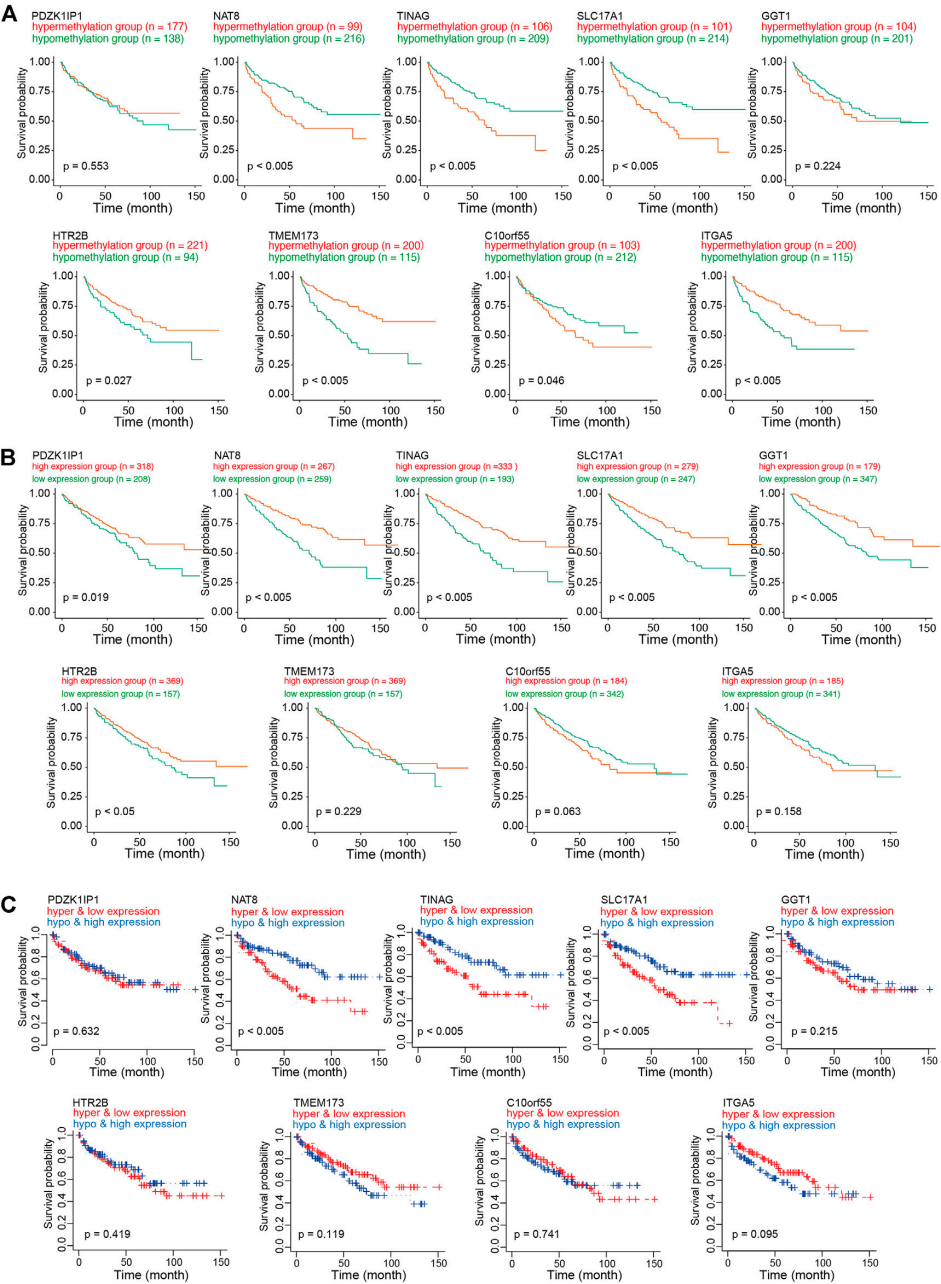
Survival analysis of CAFs related methylation-driven genes in KIRC. **(A)** Kaplan-Meier survival analysis according to methylation status of CAFs related methylation-driven genes. **(B)** Kaplan-Meier survival analysis according to RNA expression of CAFs related methylation-driven genes. **(C)** Kaplan-Meier survival analysis of methylation status combine with RNA expression of CAFs related methylation-driven genes.

To investigate the role of CAFs-related methylation-driven genes on the survival of patients with KIRC, Kaplan-Meier survival analysis was performed using DNA methylation status of CAFs-related methylation-driven genes. Survival in high methylation and low methylation group was compared using the log-rank test to determine whether the difference was significant. As shown in Figure 4A, we observed significant differences in survivals according to the methylation status of NAT8, TINAG, SLC17A1, HTR2B, TMEM173, C10orf55, SLC17A1, and GGT1. The hypermethylation status of NAT8, TINAG, SLC17A1, and C10orf55 were accompanied by worse survival conditions of patients with KIRC. In contrast, we observed that the hypomethylation status of HTR2B, TMEM173, and ITGA5 were accompanied by worse survival conditions of patients with KIRC. We then examined the correlations between RNA expression of CAFs-related methylation-driven genes and survival of patients. As shown in Figure 4B, expression levels of PDZK1IP1, NAT8, TINAG, SLC17A1, GGT1, and HT2B were significantly positively associated with survival of patients with KIRC. Furthermore, we integrated the DNA methylation dataset with RNA expression profiling. We then assessed the correlation between methylation status/RNA expression of CAFs-related methylation-driven genes and survival of patients. Patients with hypermethylation status and low expression levels of NAT8, TINAG, and SLC17A1 demonstrated a significantly shorter survival compared with those with hypomethylation status and high expression levels (Figure 4C). These results suggest that NAT8, TINAG and SLC17A1 RNA expression levels and DNA methylation status are associated with the survival of patients with KIRC.

### Clinical Significance of CAFs-Related Methylation-Driven Genes

**FIGURE 5:**
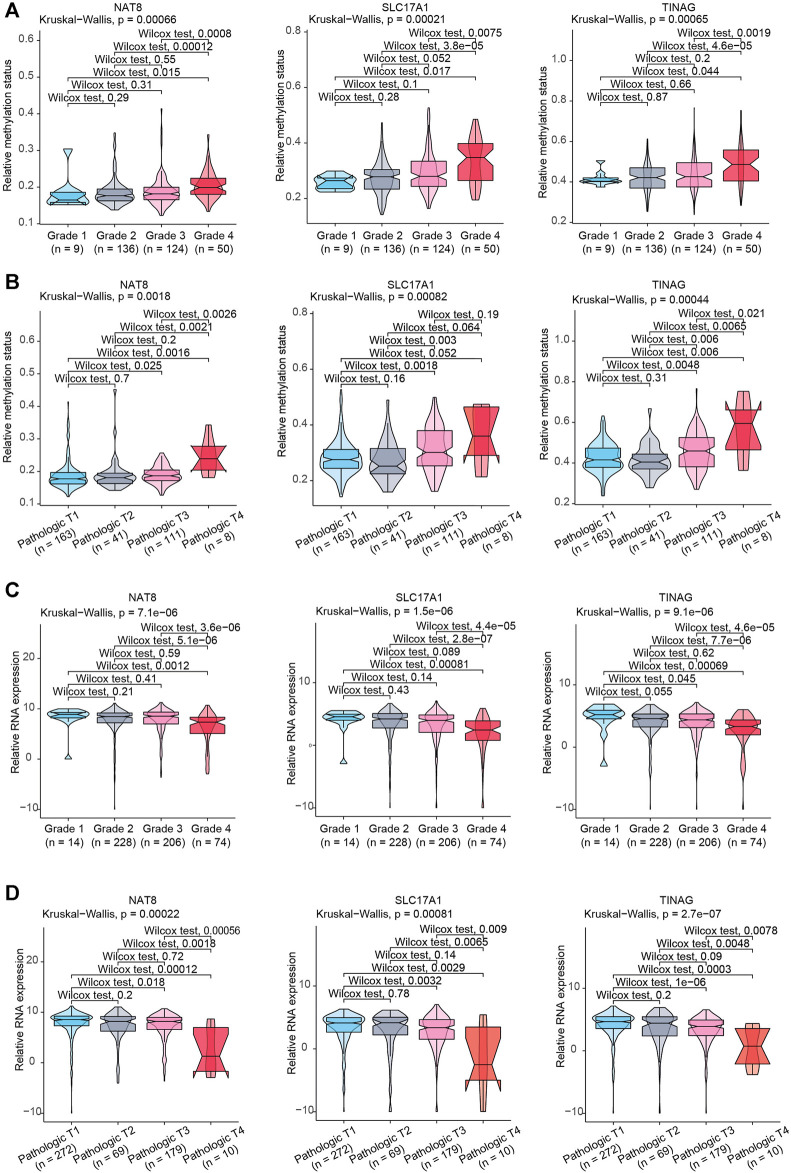
Association between clinical features and NAT8, TINAG, and SLC17A1. **(A)** Association between KIRC histologic grade and methylation status of NAT8, TINAG, and SLC17A1. **(B)** Association between KIRC pathological T grade and methylation status of NAT8, TINAG, and SLC17A1. **(C)** Association between KIRC histologic grade and RNA expression levels of NAT8, TINAG, and SLC17A1. **(D)** Association between KIRC pathological T grade and RNA expression levels of NAT8, TINAG, and SLC17A1.

To further illustrate the clinical role of NAT8, TINAG and SLC17A1, we assessed the correlations between NAT8, TINAG and SLC17A1, and clinical features. We observed that the DNA methylation levels of NAT8, TINAG and SLC17A1 were significantly associated with histologic grade and T stage of KIRC. The DNA methylation levels of NAT8, TINAG and SLC17A1were significantly higher in high-grade than in the low-grade of tumors (Figure 5A). A similar trend of correlation was observed in the T stage. As shown in Figure 5B, DNA methylation levels of NAT8, TINAG and SLC17A1 was accompanied by an increasing tumor T stage. We then examined the correlations between RNA expression levels of NAT8, TINAG and SLC17A1, and clinical features. In contrast, an opposite trend was observed for the analysis. RNA expression levels of NAT8, TINAG, and SLC17A1 were negatively correlated with histologic grade and the T stage of KIRC. Decreasing RNA expression levels of NAT8, TINAG, and SLC17A1 were associated with lower tumor grade of KIRC (Figure 5C). Meanwhile, a higher tumor T stage was accompanied by reductions of RNA expression levels of NAT8, TINAG, and SLC17A1 (Figure 5D). Taken together, We identified three clinically relevant CAFs-related methylation-driven genes containing NAT8, TINAG and SLC17A1. NAT8, TINAG, and SLC17A1 RNA expression levels and DNA methylation status are correlated with the histologic grade and T stage of patients with KIRC. We proposed that manipulation the expression of NAT8, TINAG and SLC17A1 at epigenetic level might contribute to CAFs-mediated KIRC severity.

## Discussion

In the present study, we evaluated the infiltration level of CAFs in KIRC based on a databased bioinformatic analysis. We observed the high correlations between CAFs and clinical features in KIRC. By correlating the methylation-driven genes in KIRC, the specific CAFs related methylation-driven genes were identified. Furthermore, we identified three clinically relevant CAFs-related methylation-driven genes, NAT8, TINAG, and SLC17A1. The RNA expression and methylation status of NAT8, TINAG, and SLC17A1were highly involved in clinical features of KIRC.

NAT8 belongs to the GCN5-related N-acetyltransferase superfamily (Chambers et al., 2010) that transfer the acetyl group of acetyl-coenzyme A to an acceptor substrate (Dyda et al., 2000). It has been demonstrated that NAT8 was associated with kidney disease (Luo et al., 2021; Juhanson et al., 2008). NAT8 is specifically and almost exclusively expressed in the kidney and liver (Supplementary Figure S2A) (dataset from The Human Protein Atlas) (Uhlén et al., 2015). It has been proposed that NAT8 might contribute to kidney injury via influencing acetylation pathways (Chambers et al., 2010). To date, there have been relatively few studies relevant to the biological function of NAT8 in KIRC. In particular, no relevant study identified the biological role of NAT8 in CAFs-mediated tumor microenvironment alteration. In the present study, we identified the significant correlation between NAT8 expression and CAFs infiltration level, and its clinical importance in KIRC.

TINAG encodes an extracellular matrix protein that is expressed in tubular basement membranes. Mutation of the TINAG gene is involved in nephronophthisis via influencing cell survival (Xie et al., 2011). Consistent with the previous study, we observed that TINAG is highly enriched in the kidney at both RNA and protein levels (Supplementary Figure S2B; Kanwar et al., 1999). TINAG can interact with extracellular matrix proteins (Kalfa et al., 1994; Kumar et al., 1997; Kanwar et al., 1999). It is able to regulate the adhesion of epithelial cells from kidney tubules suggesting that TINAG is crucial for communication between the cell and extracellular microenvironment (Kalfa et al., 1994; Yoshioka et al., 2002). Our knowledge of the role of TINAG is still incomplete. Particularly, the understanding of its biological function in the tumor microenvironment of KIRC is still vague. This study identified the clinical significance of the TINAG RNA expression pattern and its DNA methylation in KIRC.

SLC17A1 is the solute carrier family 17 member 1 which is expressed on the apical membrane of renal tubular cells. As the first identified member family SLC17 phosphate transporter family, SLC17A1 is proposed to mediate sodium and inorganic phosphate co-transport and believed to be the voltage-driven organic anions transporter which is highly involved in renal insufficiency (Busch et al., 1996; Hollis-Moffatt et al., 2012; Iharada et al., 2010). However, the role of SLC17A1 in the tumor microenvironment is still unknown.

Various therapeutic strategies concentrating on the tumor microenvironment are being developed and implemented in recent years. Indeed, the clinical trials are already showing promising results in KIRC (Nunes-Xavier et al., 2019). Starting from the importance of CAFs in the tumor microenvironment, the analyses of our study explored the potential biomarkers involving CAFs infiltration. In particular, we identified a set of clinically relevant CAFs-related methylation-driven genes. We are not only aim to explore the biomarkers for CAFs infiltration but also more interested in its regulation at the epigenetic level. In conclusion, our study offers a layer of CAFs regulation at the epigenetic level that can change tumor microenvironment, also may provide a therapeutic strategy to affect CAFs infiltration via manipulating clinical relevant CAFs-related methylation-driven genes in KIRC.

We performed the whole analysis based on TCGA dataset, therefore, the limitations of TCGA database should be taken into account. A limitation of the study is the relatively small amount of the cohort of KIRC dataset in TCGA database. Another limitation is that the TCGA used the Illumina 450 k BeadChip array which interrogates only about 450,000 CpG dinucleotides which only partially contains the CpGs in human genome. The incomplete coverage of the data would significantly restrict the relevant epigenetic analysis and a big amount of information of modified genes are missed when we combined the gene expression dataset and DNA methylation dataset. This might result in an analysis bias. Therefore, an improvement is urgently needed to develop analysis strategy to better use the datasets.

## Data Availability

The original contributions presented in the study are included in the article/Supplementary Material, further inquiries can be directed to the corresponding authors.
