# Supplementary figures and images for: Promising Epigenetic Biomarkers Associated With Cancer-Associated-Fibroblasts for Progression of Kidney Renal Clear Cell Carcinoma
Source: Front Genet. 2021 Sep 23;12:736156. doi: 10.3389/fgene.2021.736156 (PMC8495159; doi:10.3389/fgene.2021.736156)

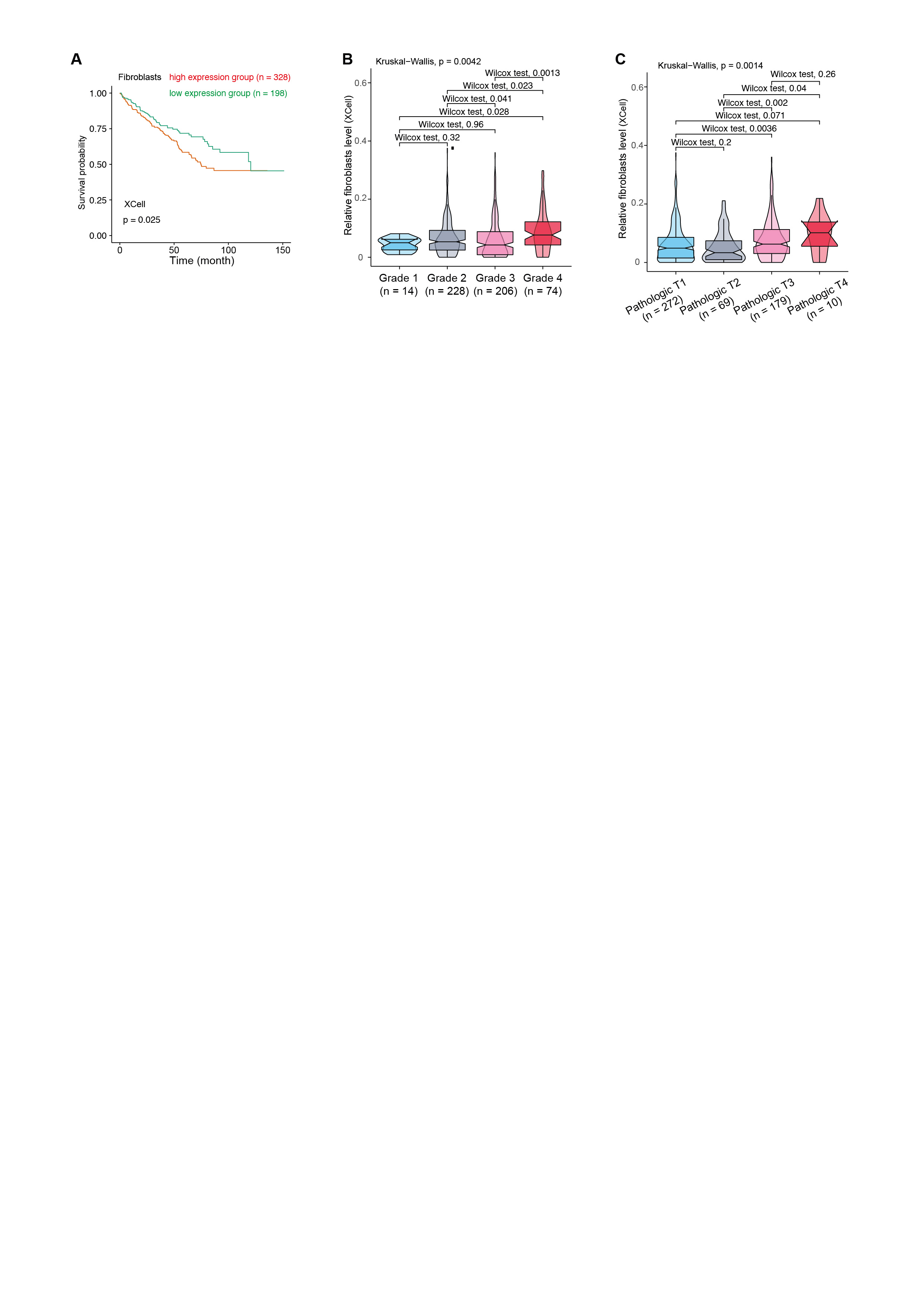

Supplement: Supplementary file 1 [file Image1.JPEG]

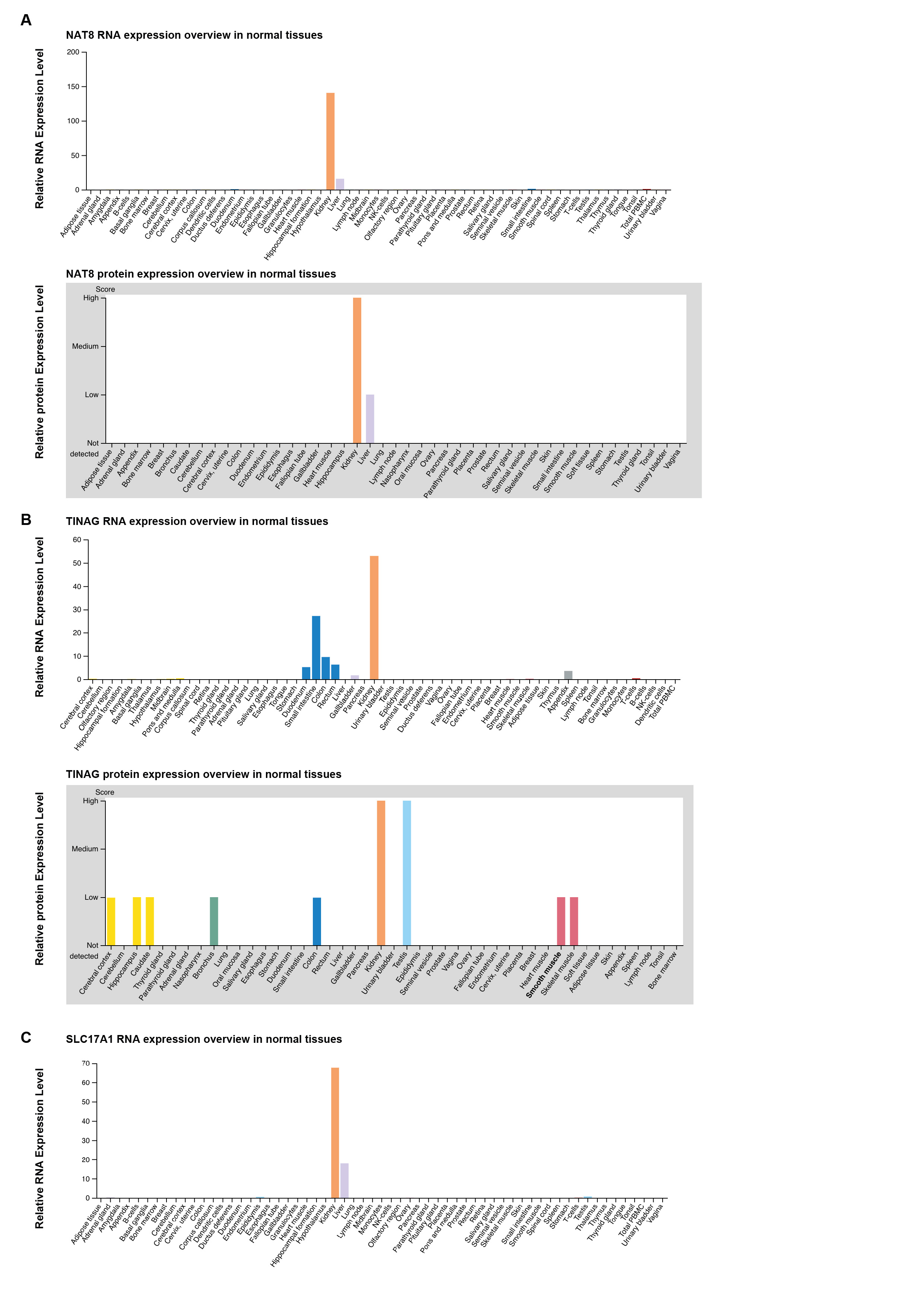

Supplement: Supplementary file 2 [file Image2.JPEG]
